# Supplementary figures and images for: Climate change as a driver of insect invasions: Dispersal patterns of a dragonfly species colonizing a new region
Source: PLoS One. 2023 Sep 14;18(9):e0291270. doi: 10.1371/journal.pone.0291270 (PMC10501572; doi:10.1371/journal.pone.0291270)

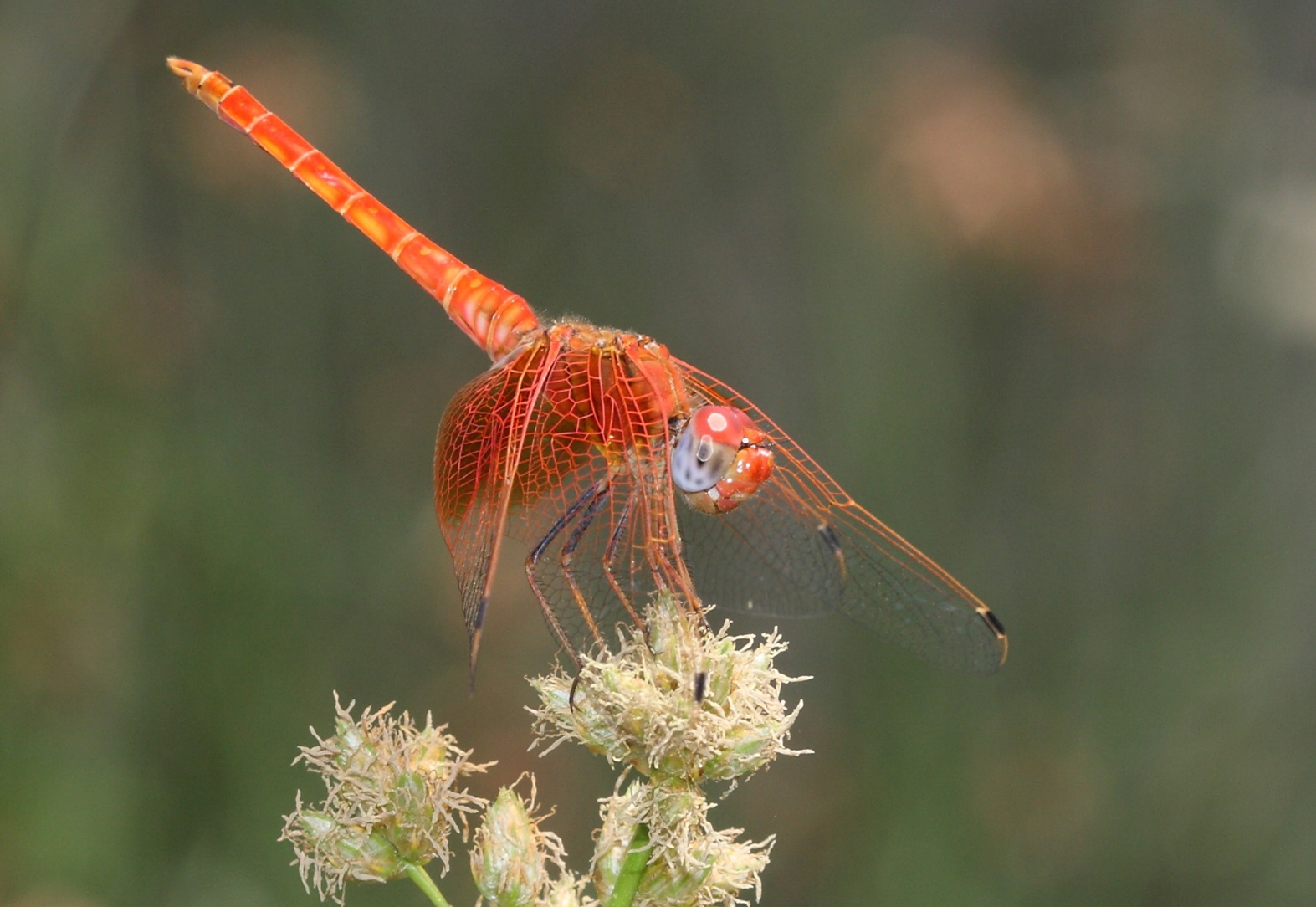

Supplement: S1 Fig — Photo credit: Jose Ignacio Pascual, with the author’s permission. Reprinted from Biodiversidad Virtual (https://www.biodiversidadvirtual.org/insectarium/Trithemis-kirbyi-Selys-1891-img769288.html) under a CC BY license, with permission from Biodiversidad Virtual, original copyright José Ignacio Pascual, 2015. (TIF) [file pone.0291270.s001.tif]

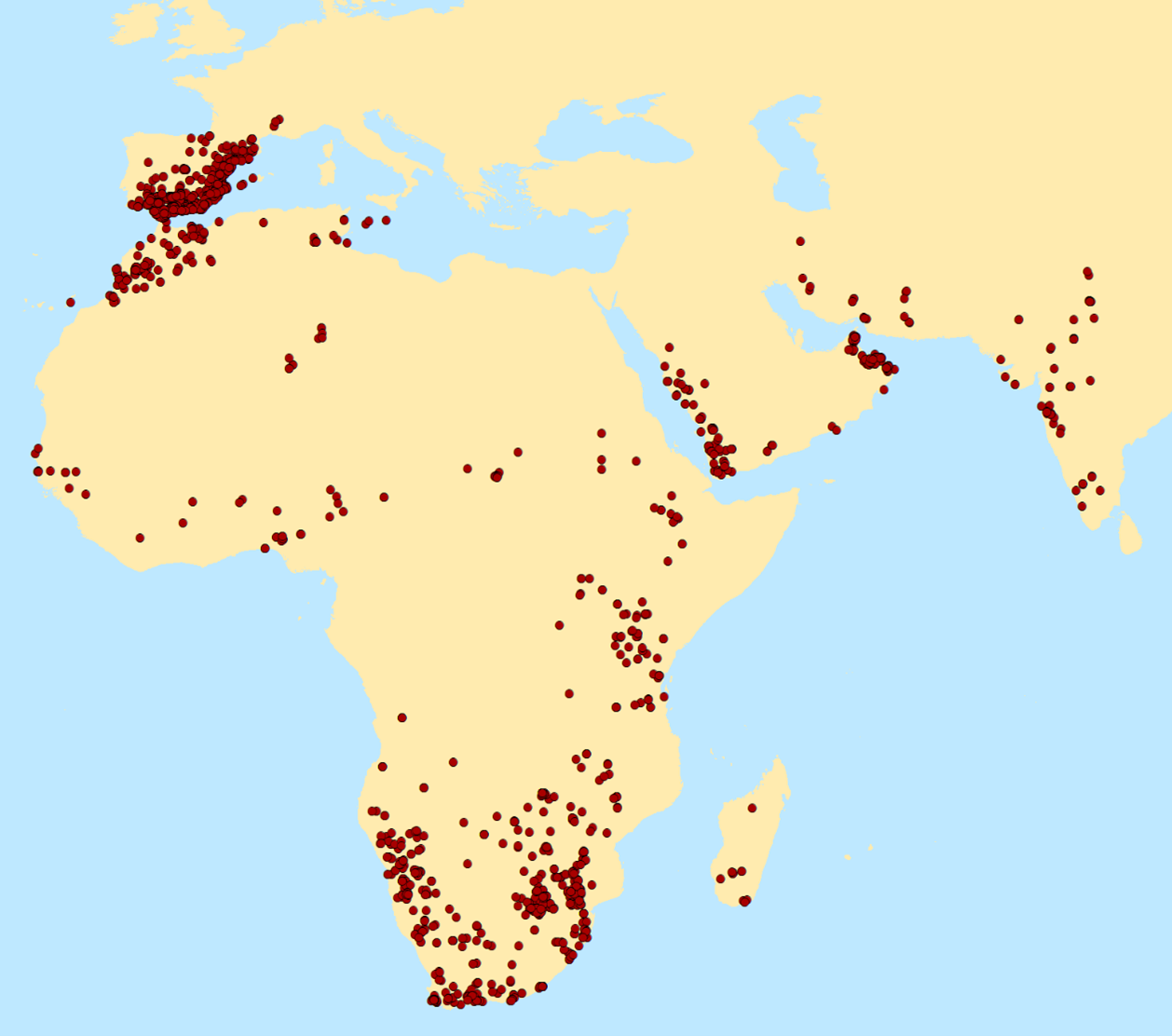

Supplement: S2 Fig — Software: ArcGIS 10.8. (TIF) [file pone.0291270.s002.tif]

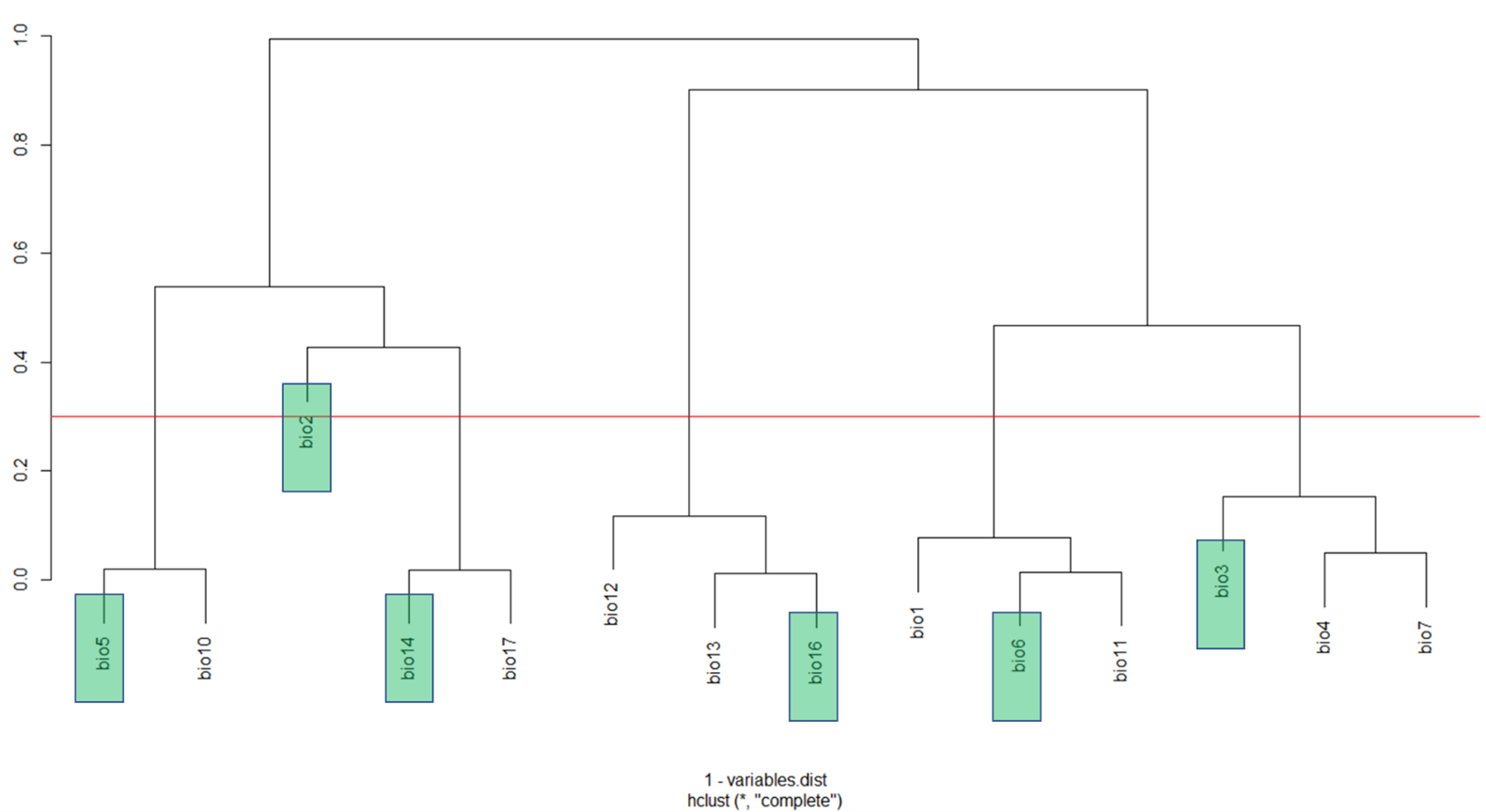

Supplement: S3 Fig — (TIF) [file pone.0291270.s003.tif]

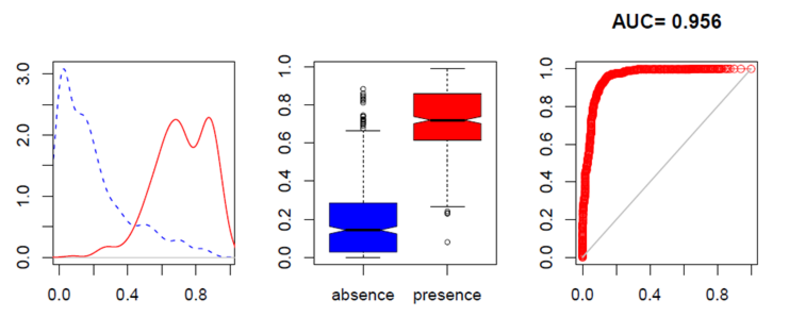

Supplement: S4 Fig — Left: Accumulation of the number of presences (red continuous line) and pseudoabsences (blue dashed line) (in y-axis) considering the suitability of the ensemble model of Trithemis kirbyi without the population density variable (in x-axis). Center: Boxplots of presences (in red) and pseudoabsences (in blue) considering the suitability of the ensemble model of Trithemis kirbyi without the population density variable (in y-axis). Right: Representation of the AUC and AUC value of the Trithemis kirbyi ensemble model without the population density variable. Software: RStudio 1.1.453, under R programming language version 3.5.0. (TIF) [file pone.0291270.s004.tif]

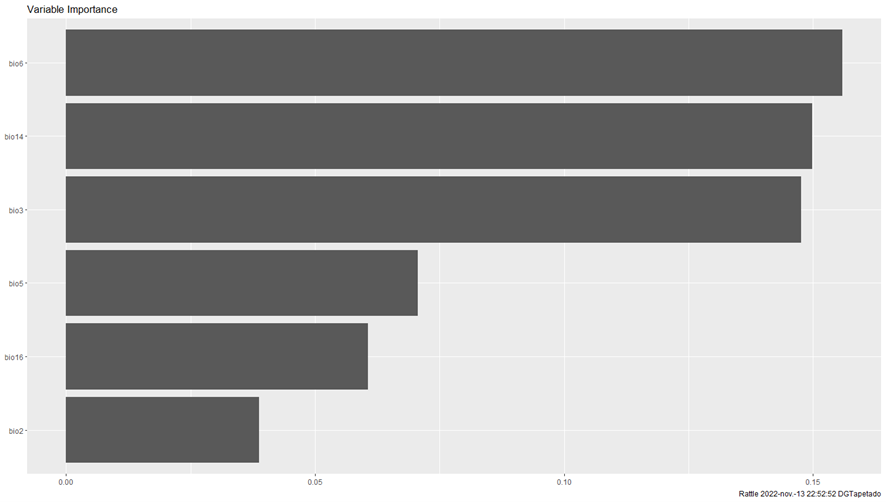

Supplement: S5 Fig — Software: RStudio 1.1.453, under R programming language version 3.5.0. (TIF) [file pone.0291270.s005.tif]

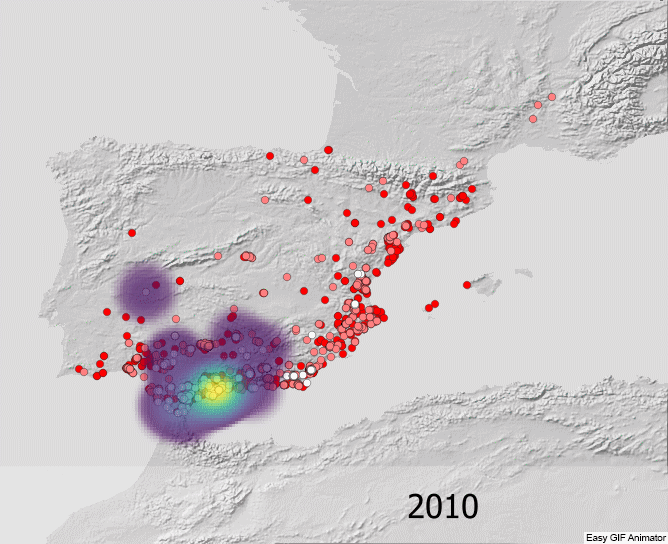

Supplement: S6 Fig — Evolution of colonization in the Iberian Peninsula between 2010 and spring 2022. The color of the collection point indicates the relative time since sites were sampled with white dots representing the oldest sites and dark red dots, the most recent ones. The heat map was generated exclusively with the presences of the year represented, and indicates a higher concentration of presences collected in the year in question. Software: ArcGIS 10.8. DEM source: USGS EROS Archive—Digital Elevation—Shuttle Radar Topography Mission (SRTM) Void Filled. Available from: https://earthexplorer.usgs.gov/. Animation generated at https://www.easygifanimator.net/. (GIF) [file pone.0291270.s006.gif]
