# Supplementary material for: Climate change as a driver of insect invasions: Dispersal patterns of a dragonfly species colonizing a new region
Source: PLoS One. 2023 Sep 14;18(9):e0291270. doi: 10.1371/journal.pone.0291270 (PMC10501572; doi:10.1371/journal.pone.0291270)
Supplement: S1 Appendix — (PDF) [file pone.0291270.s010.pdf]

```
#
~~~~~~~~~~~~~~~~~~~~~~~~~~~~~~~~~~~~~~~~~~~~~~~~~~~~~~~~~~~~~~~~~~~~~~~~~~~~~~~~~~~~~~
#

#
~~~~~~~~~~~~~~~~~~~~~~~~~~~~~~~~~~~~~~~~~~~~~~~~~~~~~~~~~~~~~~~~~~~~~~~~~~~~~~~~~~~~~~
#

# ~~~~~ Trithemis kirbyi species modelling across Iberian Peninsula~~~~~~~~ #

#
~~~~~~~~~~~~~~~~~~~~~~~~~~~~~~~~~~~~~~~~~~~~~~~~~~~~~~~~~~~~~~~~~~~~~~~~~~~~~~~~~~~~~~
#

#
~~~~~~~~~~~~~~~~~~~~~~~~~~~~~~~~~~~~~~~~~~~~~~~~~~~~~~~~~~~~~~~~~~~~~~~~~~~~~~~~~~~~~~
#

## Date: 12.2022

## Place: Madrid, SP

## Project: Trithemis kirbyi species modelling across Africa and Europe


## 0. Set up a function to install and load multiple R packages.

# Check to see if packages are installed. Install them if they are not, then load them into the R
session.

ipak <- function(pkg){

  new.pkg <- pkg[!(pkg %in% installed.packages()[, "Package"])]

  if (length(new.pkg))

    install.packages(new.pkg, dependencies = TRUE)

  sapply(pkg, require, character.only = TRUE)

}

# List of packages

packages <- c("sp","raster","dismo","maptools","rgdal","proj4","ggplot2","gridExtra",

              "cowplot","biomod2","rJava","pROC","matrixStats","usdm","kernlab",


"ks","sm","gbm","mgcv","nlme","Metrics","tidyr","readxl","RStoolbox","psych","vegan")

ipak(packages)
```

```
#
~~~~~~~~~~~~~~~~~~~~~~~~~~~~~~~~~~~~~~~~~~~~~~~~~~~~~~~~~~~~~~~~~~~~~~~~~~~~~~~
#

# ~~~~~~~~~~~~~~ Preparing the data for Trithemis kirbyi ~~~~~~~~~~~~~~~~~~~ #

#
~~~~~~~~~~~~~~~~~~~~~~~~~~~~~~~~~~~~~~~~~~~~~~~~~~~~~~~~~~~~~~~~~~~~~~~~~~~~~~~
#


## 1. Set up the pathways to access to the source of data (spatial and others)


path <- "C:/Users/DGTapetado/Desktop/Modelos2019/Modelos2022/2/"

path_wd <- "C:/Users/DGTapetado/Desktop/Modelos2019/Modelos2022/2/Kirbyi_modelling"

path_shapefiles <-
"C:/Users/DGTapetado/Desktop/Modelos2019/Modelos2022/2/General_Boundaries"

path_covariates <-
"C:/Users/DGTapetado/Desktop/Modelos2019/Modelos2022/2/Rasters_final"

path_data <- "C:/Users/DGTapetado/Desktop/Modelos2019/Modelos2022/2/Tables"

path_outputs <- "C:/Users/DGTapetado/Desktop/Modelos2019/Modelos2022/2/Kirbyi"


## 2. Import list of occurrences for Trithemis kirbyi


list.files(path_data)


setwd(path_data)

excel_sheets("Kirbyi_presencias2.xlsx")


Kirbyi.pres <- read_excel("Kirbyi_presencias2.xlsx", sheet = "75_Clim")

str(Kirbyi.pres)


Kirbyi.pres$OBJECTID<-NULL

str(Kirbyi.pres)


## 3. Create a spatial layer with the occurrences of Trithemis kirbyi for exploring geographical
incosistencies
```

```
Kirbyi.pres <- Kirbyi.pres[!is.na(Kirbyi.pres$Longitude),] # 0 records dropped out of 863

Kirbyi.pres.sp <- SpatialPoints(Kirbyi.pres[,c("Longitude","Latitude")],
                            proj4string = CRS("+proj=longlat +datum=WGS84"))

Kirbyi.pres.ly <- SpatialPointsDataFrame(Kirbyi.pres.sp, Kirbyi.pres)


## 4. Import an African map to display occurrence records of A. dorus

setwd(path_shapefiles)
list.files()
WP_ADM0 <- readOGR(dsn=path_shapefiles, layer="WPal+Afr")

setwd(path_wd)

## 5. Reproject point data to the projected coordinate system of Africa layer

PCS <- WP_ADM0@proj4string
Kirbyi.pres.ly <- spTransform(Kirbyi.pres.ly, PCS)

## 6. Remove occurrence records that do not fall within mainland

Kirbyi.pres.ly <- Kirbyi.pres.ly[WP_ADM0,] # 1 occurrence record fall out of WP mainland
Kirbyi.pres.sp <- as(Kirbyi.pres.ly, "SpatialPoints")

## 7. Identify potentially duplicated occurrences (same longitude and) and remove them
```

```
dups <- duplicated(Kirbyi.pres.ly@data[,c("Longitude","Latitude")])

length(which(dups== TRUE)) # 0 duplicated locations (based on coordinates)

Kirbyi.pres.ly <- Kirbyi.pres.ly[!dups,]
dim(Kirbyi.pres.ly) #   unique occurrences

rm(dups)
#used extent(xmin -20, xmax 55, ymin -36, ymax 40)
plot(t)
## 8. Export and save the maps
plot(WP_ADM0, xlim=c(-10,5), ylim=c(35,45),
    axes=TRUE, col="light yellow")
plot(Kirbyi.pres.ly, cex=0.5, col="red", add = TRUE)

plot(WP_ADM0,
    axes=TRUE, col="light yellow")
plot(Kirbyi.pres.ly, cex=0.5, col="red", add = TRUE)



setwd(path_outputs)
dir.create("Kirbyi.pres")
setwd("./Kirbyi.pres")

dev.print(file="Kirbyi.pres.png", device=png, width=900)
dev.off()



#
~~~~~~~~~~~~~~~~~~~~~~~~~~~~~~~~~~~~~~~~~~~~~~~~~~~~~~~~~~~~~~~~~~~~~~~~~~~~~~~~~~~~~
#
# ~~~~~~~~~~~~~~~~~~~~~~~~~~~~~~ Variable selection ~~~~~~~~~~~~~~~~~~~~~~~~~~~~~~ #
```

```
#
~~~~~~~~~~~~~~~~~~~~~~~~~~~~~~~~~~~~~~~~~~~~~~~~~~~~~~~~~~~~~~~~~~~~~~~~~~~~~~~~~~~~~
#

## 1. Load the covariates that have been prepared for this modelling exercise

# Read the path to the files with a tif extension
raster.files <- list.files(path_covariates, pattern="*tif$", full.names=TRUE)
stopifnot(length(raster.files)>0)


# Generate a stack object with all WorldClim2 dataset

Covariates_km <- stack(raster.files)
names(Covariates_km)

# Extract covariates values for the locations recording Cx pipiens

Kirbyi.pres.cov <- raster::extract(Covariates_km, Kirbyi.pres.ly)
Kirbyi.pres.cov.na <- na.omit(Kirbyi.pres.cov) # Drop 59 records because of the presence of null values

## 6. Final selected covariates

names(Covariates_km)
Predictors <- Covariates_km
names(Predictors)


#latlon <- "+proj=longlat +datum=WGS84"
#Predictors <- projectRaster(Predictors, crs=latlon)
```

```
#
~~~~~~~~~~~~~~~~~~~~~~~~~~~~~~~~~~~~~~~~~~~~~~~~~~~~~~~~~~~~~~~~~~~~~~~~~~~~~~~~~~~~~~
#

# ~~~~~~~~~~~~~~ Generate pseudo-absences and background points ~~~~~~~~~~~~~~~ #

#
~~~~~~~~~~~~~~~~~~~~~~~~~~~~~~~~~~~~~~~~~~~~~~~~~~~~~~~~~~~~~~~~~~~~~~~~~~~~~~~~~~~~~~
#


## We want to generate background points and pseudo-absences together using the following criteria:

# For background points, we will account for geographical bias on presence distribution.

# For pseudo-absences points, we will generate points from areas that we certainly know to be unsuitable

# for the presence of mosquitoes: arids and extreme arid areas, and above certain altitude.


## 1. Load in custom functions


setwd(path_wd)

source("SEEG_SDM.R")


setwd(path_data)

list.files(path_data)

# 2.1. Raster object with AI and SRTM


absences.df <- read_excel("Kirbyi_pseudoausencias2.xlsx", sheet = "75_Clim")

str(absences.df)

absences.df$ID<-NULL

absences.df$Scx<-0

str(absences.df)


absences.df_coords<-absences.df[,1:2]

absences.df_Scx<-absences.df[,9]
```

```
absences.df_Scx<-as.data.frame(absences.df_Scx)


absences.ly <- SpatialPointsDataFrame(absences.df_coords, absences.df_Scx, proj4string =
PCS)

c<-as.data.frame(absences.ly)


x11()

plot(WP_ADM0)

plot(absences.ly, cex = 0.5, col = "red", add = T)



## 3. Generate background points

list.files(path_data)

BCG.bias<- read_excel("Kirbyi_background2.xlsx", sheet = "Kirbyi_background")

str(BCG.bias)

BCG.bias$ID<-NULL

BCG.bias$Scx<-0

BCG.sp<-BCG.bias[,c(1,2)]

ScxPPS<-BCG.bias[,9]

ScxPPS<-as.data.frame(ScxPPS)

BCG.bias <- SpatialPointsDataFrame(BCG.sp, ScxPPS, proj4string = PCS)


x11()

plot(WP_ADM0)

plot(BCG.bias, cex = 0.5, col = "red", add = T)


## 4. Generate final presence dataset for Trithemis kirbyi

# 4.1. Extract true absences from nation-wide survey, which is more reliable


str(Kirbyi.pres)

Kirbyi.pres$Scx<-1
```

```
Kirbyi.pres.sp<-Kirbyi.pres[,c(1,2)]

Kirbyi.pres.PPS<-Kirbyi.pres[,9]

Kirbyi.pres.PPS<-as.data.frame(Kirbyi.pres.PPS)


Kirbyi.pres.final <- SpatialPointsDataFrame(Kirbyi.pres.sp, Kirbyi.pres.PPS, proj4string = PCS)


###HASTA AQU?###




## 5. Create a weight for each background point set it so that the weight of the absence and
pseudo-absence records sums to the weight of the presence points to improve model fitting


npres <- nrow(Kirbyi.pres.final)+1 # 862

nabs <- nrow(absences.ly) # 400

nbcg <- nrow(BCG.bias) # 800


npres

nabs

nbcg



bg_weight <- abs((npres - nabs) / nbcg)

#(416-187)/600


# 5.1. Create a vector of regression weights


weights <- c(rep(1, npres), rep(0.5, nabs), rep(bg_weight, nbcg))



# 5.2. Merge Presence, pseudo-absences and background datasets
```

```
Kirbyi.pres.pps <- spRbind(Kirbyi.pres.final, absences.ly)

Kirbyi.pres.pps <- spRbind(Kirbyi.pres.pps, BCG.bias)

# 5.3. Export as shp file the Occurrence point layer

writeOGR(Kirbyi.pres.pps, dsn = path_outputs, layer ="Kirbyi.pres",
        driver = "ESRI Shapefile", overwrite_layer = T)

#
~~~~~~~~~~~~~~~~~~~~~~~~~~~~~~~~~~~~~~~~~~~~~~~~~~~~~~~~~~~~~~~~~~~~~~~~~~~~~~~~~~~~~~
#
# ~~~~~~~~~~~~~~~~ Running Distribution Modelling: using Biomod ~~~~~~~~~~~~~~~~ #
#
~~~~~~~~~~~~~~~~~~~~~~~~~~~~~~~~~~~~~~~~~~~~~~~~~~~~~~~~~~~~~~~~~~~~~~~~~~~~~~~~~~~~~~
#

## 0. Cross-check for potential NA values that can make the modelling run crashed out.

Kirbyi.pres.pps.pred <- raster::extract(Predictors, Kirbyi.pres.pps)

nl <- which(complete.cases(Kirbyi.pres.pps.pred)) # 10 records have some nulls values for the
predictors.

Kirbyi.pres.pps <- Kirbyi.pres.pps[nl,] # keep those records with completed values
weights <- weights[nl] # from list of weights, remove those with no NA values

## 1. Compiling the different elements to be using when computing models

setwd(path_wd)

Predictors1 <- stack(Predictors)
```

```
#("PET","MaxTempWM","MinTempCM","DistWB","DistRivers","FlowAcc","LandUses")


Kirbyi.pres.pps.Formatted <- BIOMOD_FormatingData(resp.var = Kirbyi.pres.pps, expl.var = 
Predictors1, resp.name = "Kirbyi.presPPS")


## 2. Modelling. Building models based on the following algorithms: 
GLM,GAM,SRE,ANN,CTA,RF,MAXENT


path_maxent <- 
"C:/Users/DGTapetado/Desktop/Modelos2019/Modelos2022/1/Kirbyi_modelling/MaxEnt"

list.files(path_maxent)

jar <- paste(path_maxent, "maxent.jar", sep='/')


myBiomodOption <- BIOMOD_ModelingOptions(MAXENT.Phillips = list(path_to_maxent.jar = 
jar))


system.time(Kirbyi.pres.model1 <- BIOMOD_Modeling(Kirbyi.pres.pps.Formatted,

                          models = c("GLM","ANN", "CTA",

                                 "RF", "MAXENT.Phillips", "GAM"),

                          models.options = myBiomodOption,

                          NbRunEval = 2,

                          DataSplit = 85,

                          Yweights = weights,

                          VarImport = 1,

                          models.eval.meth = c("TSS","ROC","ACCURACY"),

                          SaveObj = TRUE,

                          rescal.all.models = FALSE,

                          do.full.models = FALSE,

                          modeling.id = paste("Kirbyi.presPPS","Final",sep =".")))
```

```
## system elapsed: 3971.16  (66.7 min)

Kirbyi.pres.model1 ## None of the models failed

# 2.1. Extract evaluations from all models run
Kirbyi.pres.model1.Eval <- get_evaluations(Kirbyi.pres.model1)
dimnames(Kirbyi.pres.model1.Eval)

## Visualize performance of the models

setwd(path_outputs)
setwd("./Kirbyi.pres")

theme_set(theme_grey())
performance.models <- models_scores_graph(Kirbyi.pres.model1, by = "models",
                                metrics = c("ROC","TSS"))

dev.print(file="Performance_Kirbyi.pres_models.png", device=png, width=900)
dev.off()

performance.models.1 <- models_scores_graph(Kirbyi.pres.model1, by = "models",
                                 metrics = c("ROC","ACCURACY"))

dev.print(file="Performance_Kirbyi.pres_models_models2.png", device=png, width=900)
dev.off()


# TSS/ROC/ACCURACY for test data (TSS: True Skill Statistic)

TSS.test.data <- as.data.frame(t(Kirbyi.pres.model1.Eval["TSS","Testing.data",,,]))
ROC.test.data <- as.data.frame(t(Kirbyi.pres.model1.Eval["ROC","Testing.data",,,]))
```

```
PCC.test.data <- as.data.frame(t(Kirbyi.pres.model1.Eval["ACCURACY","Testing.data",,,]))

summary(TSS.test.data)
summary(ROC.test.data)
summary(PCC.test.data)

TSS.Mean <- apply(TSS.test.data, 2, mean, na.rm = T)
TSS.Median <- apply(TSS.test.data, 2, median, na.rm = T)
TSS.1Q <- apply(TSS.test.data, 2, function (x) quantile(x, c(0.25), type = 7, na.rm = T))
TSS.3Q <- apply(TSS.test.data, 2, function (x) quantile(x, c(0.75), type = 7, na.rm = T))

TSS <- t(data.frame(TSS.Mean, TSS.Median, TSS.1Q, TSS.3Q))

ROC.Mean <- apply(ROC.test.data, 2, mean, na.rm = T)
ROC.Median <- apply(ROC.test.data, 2, median, na.rm = T)
ROC.1Q <- apply(ROC.test.data, 2, function (x) quantile(x, c(0.25), type = 7, na.rm = T))
ROC.3Q <- apply(ROC.test.data, 2, function (x) quantile(x, c(0.75), type = 7, na.rm = T))

ROC <- t(data.frame(ROC.Mean, ROC.Median, ROC.1Q, ROC.3Q))

PCC.Mean <- apply(PCC.test.data, 2, mean, na.rm = T)
PCC.Median <- apply(PCC.test.data, 2, median, na.rm = T)
PCC.1Q <- apply(PCC.test.data, 2, function (x) quantile(x, c(0.25), type = 7, na.rm = T))
PCC.3Q <- apply(PCC.test.data, 2, function (x) quantile(x, c(0.75), type = 7, na.rm = T))

PCC <- t(data.frame(PCC.Mean, PCC.Median, PCC.1Q, PCC.3Q))

write.csv(TSS, file = "Kirbyi.pres_TSS.csv")
write.csv(ROC, file = "Kirbyi.pres_ROC.csv")
write.csv(PCC, file = "Kirbyi.pres_PCC.csv")
```

```
rm(TSS.Mean, TSS.Median, TSS.1Q, TSS.3Q, ROC.Mean, ROC.Median, ROC.1Q, ROC.3Q,
   PCC.Median, PCC.Mean, PCC.1Q, PCC.3Q)

# 2.2. Get variable importance for the different models

Kirbyi.pres.model1.Var <- get_variables_importance(Kirbyi.pres.model1)
dimnames(Kirbyi.pres.model1.Var)

Variable.Contribution <- t(apply(Kirbyi.pres.model1.Var, 2, rowMeans, na.rm = T))

write.csv(Variable.Contribution, file = "Variable_Contribution_Kirbyi.pres.csv")

setwd(path_wd)

# 2.3. Explore variable response curves for RF

Kirbyi.pres_RF <- BIOMOD_LoadModels(Kirbyi.pres.model1, models = "RF")

RFsPlot2D <- response.plot2(models = Kirbyi.pres_RF,
                  Data = get_formal_data(Kirbyi.pres.model1,"expl.var"),
                  show.variables = get_formal_data(Kirbyi.pres.model1, "expl.var.names"),
                  do.bivariate = FALSE,
                  fixed.var.metric = "median",
                  col = c("blue", "red"),
                  legend = TRUE,
                  data_species = get_formal_data(Kirbyi.pres.model1, "resp.var"))



## 3. Ensemble Modelling. Come up with a consensus model and ensemble for every type of
model
```

```
Kirbyi.pres.model1.Ensemble <- BIOMOD_EnsembleModeling(modeling.output =
Kirbyi.pres.model1,
                                            chosen.models = "all",
                                            em.by = "all",
                                            eval.metric = c("ROC"),
                                            eval.metric.quality.threshold = c(0.70),
                                            prob.mean = T,
                                            prob.cv = T,
                                            prob.ci = T,
                                            prob.ci.alpha = 0.05,
                                            prob.median = T,
                                            committee.averaging = T,
                                            prob.mean.weight = T,
                                            prob.mean.weight.decay = "proportional")

Kirbyi.pres.model1.Ensemble

# 3.1. Extract evaluation of the ensemble model

Ensemble.Algorithms <-
c("Mean","Coef.Var","InfCI","SupCI","Median","MCAvg","WeightedMean")

Kirbyi.pres.Ensemble.Eval <- get_evaluations(Kirbyi.pres.model1.Ensemble)

Kirbyi.pres.Ensemble.TSS <- t(sapply(Kirbyi.pres.Ensemble.Eval, "[",2,1:4))
rownames(Kirbyi.pres.Ensemble.TSS) <- Ensemble.Algorithms

Kirbyi.pres.Ensemble.KAPPA <- t(sapply(Kirbyi.pres.Ensemble.Eval, "[",1,1:4))
rownames(Kirbyi.pres.Ensemble.KAPPA) <- Ensemble.Algorithms

Kirbyi.pres.Ensemble.ROC <- t(sapply(Kirbyi.pres.Ensemble.Eval, "[",3,1:4))
```

```
rownames(Kirbyi.pres.Ensemble.ROC) <- Ensemble.Algorithms


setwd(path_outputs)

setwd("./Kirbyi.pres")


write.csv(Kirbyi.pres.Ensemble.TSS, file = "Kirbyi.pres_Ensemble_TSS.csv")

write.csv(Kirbyi.pres.Ensemble.KAPPA, file = "Kirbyi.pres_Ensemble_KAPPA.csv")

write.csv(Kirbyi.pres.Ensemble.ROC, file = "Kirbyi.pres_Ensemble_ROC.csv")


setwd(path_wd)


## 4. Final projection (extrapolation) based on the best fitted model.

# 4.1. Space projection for the algorithms selected


system.time(Kirbyi.pres.Proj <- BIOMOD_Projection(modeling.output = Kirbyi.pres.model1,
new.env = Predictors1, proj.name = "Final", selected.models = "all", binary.meth = "TSS",
compress = "gzip", build.clamping.mask = FALSE, output.format = ".grd"))



## system elapsed: 38812.16 (10.8 h)


Kirbyi.pres.Proj

list.files("Kirbyi.presPPS/proj_Final")


Kirbyi.pres.projections <- get_predictions(Kirbyi.pres.Proj) # Set up a raster stack with the
predictions



## 5. Ensemble forescasting combining projections based on models ensemble rules defined at
the ensemble modelling step

Kirbyi.pres.EF <- BIOMOD_EnsembleForecasting(EM.output = Kirbyi.pres.model1.Ensemble,

                              projection.output = Kirbyi.pres.Proj)
```

```
Kirbyi.pres.Ensemble <- get_predictions(Kirbyi.pres.EF)

names(Kirbyi.pres.Ensemble)

# Extract best performing models: mean of probabilities with CI and models committe averaging

Kirbyi.pres.mean <- raster(Kirbyi.pres.Ensemble, layer=1)/1000
Kirbyi.pres.LB <- raster(Kirbyi.pres.Ensemble, layer=3)/1000
Kirbyi.pres.UB <- raster(Kirbyi.pres.Ensemble, layer = 4)/1000
Kirbyi.pres.MCA <- raster(Kirbyi.pres.Ensemble, layer = 6)/1000
Kirbyi.pres.WMP <- raster(Kirbyi.pres.Ensemble, layer = 7)/1000


par(mfrow=c(1,3))
plot(Kirbyi.pres.LB)
plot(Kirbyi.pres.WMP)
plot(Kirbyi.pres.UB)

par(mfrow=c(1,1))

## 6. Export resulting raster datasets

setwd(path_outputs)
setwd("./Kirbyi.pres")


writeRaster(Kirbyi.pres.mean,filename = "./Kirbyi.pres_Mean.tif", format= "GTiff", overwrite=TRUE)
writeRaster(Kirbyi.pres.LB,filename = "./Kirbyi.pres_LB.tif", format= "GTiff", overwrite=TRUE)
```

```
writeRaster(Kirbyi.pres.UB,filename = "./Kirbyi.pres_UB.tif", format= "GTiff", overwrite=TRUE)

writeRaster(Kirbyi.pres.WMP,filename = "./Kirbyi.pres_WeigthedMean2.tif", format= "GTiff", overwrite=TRUE)


x11()

plot(Kirbyi.pres.mean)

plot(Kirbyi.pres.ly, cex=0.5, col="red", add = TRUE)



#install.packages("raster")

#install.packages("dismo")

#install.packages("readxl")


library(raster)

library(dismo)

library(readxl)


path_wd<-"C:/Users/DGTapetado/Desktop/Modelos2019/Modelos2022/2/Tables"

path_mod<-"C:/Users/DGTapetado/Desktop/Modelos2019/Modelos2022/2/Kirbyi/Kirbyi.pres"

path_pdf<-"C:/Users/DGTapetado/Desktop/Modelos2019/Modelos2022/2/Kirbyi/Kirbyi.pres"

setwd(path_wd)

list.files(path_wd)


presencia.evaluacion <- read_excel("Kirbyi_presencias.xlsx", sheet = "25_Clim")

str(presencia.evaluacion)

presencia.evaluacion<-presencia.evaluacion[,c(2,3)]

colnames(presencia.evaluacion)[2]<-"y"

colnames(presencia.evaluacion)[1]<-"x"

presencia.evaluacion$Scx<-1

str(presencia.evaluacion)
```

```
presencia.evaluacion.sp <- SpatialPoints(presencia.evaluacion[,c("x","y")],
                                         proj4string = CRS("+proj=longlat +datum=WGS84"))

#importa las ausencias



absences.table <- read_excel("Kirbyi_pseudoausencias.xlsx", sheet = "25_Clim")
absences.table<-absences.table[,c(2,3)]
colnames(absences.table)[2]<-"y"
colnames(absences.table)[1]<-"x"
absences.table$Scx<-0
str(absences.table)
absences<-SpatialPoints(absences.table)



setwd(path_mod)

#importa los modelos
lista.modelos2 <- list.files(path="./",pattern='.tif$', full.names=TRUE)
lista.modelos2
modelos<- brick(stack(lista.modelos2))

names(modelos)

x11()
plot(modelos)
```

```
x11()
plot(modelos[[2]])
plot(presencia.evaluacion.sp, cex = 0.5, col = "red", add = T)
plot(absences, cex = 0.5, col = "black", add = T)




evaluacion.modelo<-rbind(absences.table,presencia.evaluacion)
evaluacion.modelo2<-evaluacion.modelo
evaluacion.modelo2$Scx<-NULL
evaluacion.modelo2

#extraemos los valores de los puntos de evaluación en el modelo
valores.puntos.evaluacion<-raster::extract(modelos, evaluacion.modelo2[, c("x","y")])
str(valores.puntos.evaluacion)



#es una lista, pasamos a data.frame
valores.puntos.evaluacion2<-as.data.frame(valores.puntos.evaluacion)
str(valores.puntos.evaluacion2)

#lo unimos con la columna presencia de presencias.test
valores.puntos.evaluacion2$Scx<-evaluacion.modelo$Scx
str(valores.puntos.evaluacion2)



evaluacion.modelo <- evaluacion.modelo[!is.na(evaluacion.modelo$Scx),]
str(evaluacion.modelo)

evaluacion.modelo<-cbind(evaluacion.modelo, valores.puntos.evaluacion2)
```

```
#evaluacion.modelo<-evaluacion.modelo[,c(1,2,3,4)]

#valores.puntos.evaluacion2 <-
evaluacion.modelo[!is.na(evaluacion.modelo$valores.puntos.evaluacion),]

valores.puntos.evaluacion2<- as.data.frame(valores.puntos.evaluacion2)


#creamos una tabla en blanco para guardar los resultados

resultados.evaluacion<-data.frame(modelo=character(), auc=numeric(), cor=numeric(),
stringsAsFactors=FALSE)


#contador de filas

fila=0

valores.puntos.evaluacion<-evaluacion.modelo


setwd(path_pdf)


#EMPIEZA A EJECUTAR EL LOOP AQUÍ

#abrimos un pdf para guardar los gráficos

pdf("./evaluacion_modelos2.pdf", width=15, height=8, pointsize=30)


#iteramos por cada uno de los modelos

for (modelo in names(modelos)){


  #separamos los valores de las presencias y las backgrounds

  #f????jate como en cada iteración tomamos la columna 'modelo'

  valores.presencias<-valores.puntos.evaluacion[valores.puntos.evaluacion$Scx==1, modelo]

  valores.background<-valores.puntos.evaluacion[valores.puntos.evaluacion$Scx==0, modelo]


  #evaluamos el modelo

  evaluacion<-dismo::evaluate(p=valores.presencias, a=valores.background)


  #suma 1 a la fila de la tabla de resultados

  fila=fila+1
```

```
  #llenamos la tabla
  resultados.evaluacion[fila, "modelo"]<-modelo
  resultados.evaluacion[fila, "auc"]<-evaluacion@auc
  resultados.evaluacion[fila, "cor"]<-evaluacion@cor

  #dibujamos el gráfico
  #plots
  par(mfrow=c(1,3), mar=c(2,2,4,2), oma=c(3,3,5,3))
  density(evaluacion)
  boxplot(evaluacion, col=c("blue", "red"))
  plot(evaluacion, "ROC")
  mtext(modelo, outer=TRUE, cex=1.3)

}

dev.off()
#TERMINA DE EJECUTAR EL LOOP AQUÍ



#####Boxplots Variables#######



dir<-"C:/Users/DGTapetado/Desktop/Modelos2019/Modelos2022/2"


#<-"C:/Users/DGTapetado/Downloads"
setwd(dir)


#install.packages("wesanderson")


library(readxl)
```

```
library(ggplot2)

data <- read_excel("Table_Analyses.xlsx", sheet = "Presences_IP_new5")




a<-ggplot(data = data, aes(x=Pres_CAT, y=bio2)) + geom_boxplot(aes(fill=Pres_CAT)) +
  scale_fill_brewer(palette="Set1", direction=-1) + theme_classic() +
theme(legend.position="none") +
  labs(x = " ", y= "Mean Diurnal Range")
b<-ggplot(data = data, aes(x=Pres_CAT, y=bio3)) + geom_boxplot(aes(fill=Pres_CAT)) +
  scale_fill_brewer(palette="Set1", direction=-1) + theme_classic() +
theme(legend.position="none")+
  labs(x = " ", y= "Isothermality")
c<-ggplot(data = data, aes(x=Pres_CAT, y=bio5)) + geom_boxplot(aes(fill=Pres_CAT)) +
  scale_fill_brewer(palette="Set1", direction=-1) + theme_classic() +
theme(legend.position="none")+
  labs(x = " ", y= "Max Temperature of Warmest Month")
d<-ggplot(data = data, aes(x=Pres_CAT, y=bio6)) + geom_boxplot(aes(fill=Pres_CAT)) +
  scale_fill_brewer(palette="Set1", direction=-1) + theme_classic() +
theme(legend.position="none")+
  labs(x = " ", y= "Min Temperature of Coldest Month")
e<-ggplot(data = data, aes(x=Pres_CAT, y=bio14)) + geom_boxplot(aes(fill=Pres_CAT)) +
  scale_fill_brewer(palette="Set1", direction=-1) + theme_classic() +
theme(legend.position="none")+
  labs(x = " ", y= "Precipitation of Driest Month")
f<-ggplot(data = data, aes(x=Pres_CAT, y=bio16)) + geom_boxplot(aes(fill=Pres_CAT)) +
  scale_fill_brewer(palette="Set1", direction=-1) + theme_classic() +
theme(legend.position="none")+
  labs(x = " ", y= "Precipitation of Wettest Quarter")


g<-ggplot(data = data, aes(x=Pres_CAT, y=bio1)) + geom_boxplot(aes(fill=Pres_CAT)) +
```

```
  scale_fill_brewer(palette="Set1", direction=-1) + theme_classic() +
theme(legend.position="none")+
  labs(x = " ", y= "Annual Mean Temperature")

x11()
plot(a)
x11()
plot(b)
x11()
plot(c)
x11()
plot(d)
x11()
plot(e)
x11()
plot(f)

x11()
plot(g)

library(gridExtra)

x11()
grid.arrange(a, b, c, d, e, f, ncol=3, nrow =2)

#####Graph 4 variable interaction######
```

```
##Perform two and join in the same image#######


library(readxl)

library(ggplot2)

data <-read_excel("Data_Area-Thermal anomaly.xlsx",

                  sheet="Hoja1")




p1 <- ggplot(data, aes(Year, Area)) +

  geom_line() + theme(axis.line = element_line())

p2 <- ggplot(data, aes(Year, Thermic_anomaly)) + geom_point() +

  theme(axis.line = element_line())

p3 <- ggplot(data, aes(Year, Increase/0.5)) +

  geom_line(aes( color = "Area Increase"), size = 1,linetype = "dotdash", color= "midnightblue")
+

  geom_line(aes(y = Area, color = "Total Area"), size = 1, color= "forestgreen") +

  geom_line(aes(y = Thermic_anomaly / 0.000002), size = 1, linetype = "dashed", color =
"darkred") +

  theme(axis.line = element_line(),

        plot.margin = margin(10, 10, 10, 30))

x11()

wrap_elements(get_plot_component(p1, "ylab-l")) +

  wrap_elements(get_y_axis(p1)) +

  wrap_elements(get_plot_component(p2, "ylab-l")) +

  wrap_elements(get_y_axis(p2)) +

  p3 +

  plot_layout(widths = c(3, 1, 3, 1, 40)) + theme_classic()
```
