# Supplementary material for: Climate change as a driver of insect invasions: Dispersal patterns of a dragonfly species colonizing a new region
Source: PLoS One. 2023 Sep 14;18(9):e0291270. doi: 10.1371/journal.pone.0291270 (PMC10501572; doi:10.1371/journal.pone.0291270)
Supplement: Attachment — Submitted filename: Response to Reviewers.docx [file pone.0291270.s011.docx]

Dear Editor,

We are resubmitting our revised version of the manuscript PONE-D-23-22273 entitled ‘Climate change as a driver of insect invasions: Dispersal patterns of a dragonfly species colonizing a new region’.

We really acknowledge the useful comments and suggestions of the reviewers as well as the editorial guidelines, which have undoubtedly helped to improve the new version of our work. We revised and modified the manuscript according to the reviewers’ comments. The suggestions pointed out by the reviewers as well as by you have been implemented, and can be tracked in the “Revised Manuscript with Track Changes.docx” document.

We have also included that the species has been found recently in Belgium, supporting the arguments in our paper. The sentence reads “In fact, during the writing of this manuscript, T. kirbyi was also found in Belgium during a high temperature episode, reinforcing the idea of the importance of this type of platforms and data, as well as evaluating and validating our model (suitability of this area of Belgium = 0.6) [63].”, and one more reference has been added.

We hope you find the revised version of the manuscript acceptable for publication in *PLoS ONE*.

Thank you for your time. Best regards.

Diego Gil-Tapetado on behalf of all the authors.

1. Please ensure that your manuscript meets PLOS ONE's style requirements, including those for file naming. The PLOS ONE style templates can be found at

**•Response: We have reformatted the ms considering PLoS style.**

2. Please note that PLOS ONE has specific guidelines on code sharing for submissions in which author-generated code underpins the findings in the manuscript. In these cases, all author-generated code must be made available without restrictions upon publication of the work. Please review our guidelines at https://journals.plos.org/plosone/s/materials-and-software-sharing#loc-sharing-code and ensure that your code is shared in a way that follows best practice and facilitates reproducibility and reuse.

**•Response: We have uploaded the code as Supplementary material.**

3. We suggest you thoroughly copyedit your manuscript for language usage, spelling, and grammar. If you do not know anyone who can help you do this, you may wish to consider employing a professional scientific editing service.

Whilst you may use any professional scientific editing service of your choice, PLOS has partnered with both American Journal Experts (AJE) and Editage to provide discounted services to PLOS authors. Both organizations have experience helping authors meet PLOS guidelines and can provide language editing, translation, manuscript formatting, and figure formatting to ensure your manuscript meets our submission guidelines. To take advantage of our partnership with AJE, visit the AJE website (http://learn.aje.com/plos/) for a 15% discount off AJE services. To take advantage of our partnership with Editage, visit the Editage website (www.editage.com) and enter referral code PLOSEDIT for a 15% discount off Editage services. If the PLOS editorial team finds any language issues in text that either AJE or Editage has edited, the service provider will re-edit the text for free.

**•Response: The manuscript was edited by a native English professional editor, Dra. Melinda Modrell, who actually works as Managing Editor for the journals “Evolution” and “Evolution Letters” (https://www.evolutionsociety.org/news/display/2022/11/29/welcome-new-evolution-and-evolution-letters-managing-editor-melinda-modrell/)**

Upon resubmission, please provide the following:

● The name of the colleague or the details of the professional service that edited your manuscript

**•Response:** **Dra. Melinda Modrell (mmodrell@gmail.com)**

● A copy of your manuscript showing your changes by either highlighting them or using track changes (uploaded as a *supporting information* file)

**•Response: Done**

● A clean copy of the edited manuscript (uploaded as the new *manuscript* file)

**•Response: Done**

4. Please note that funding information should not appear in any section or other areas of your manuscript. We will only publish funding information present in the Funding Statement section of the online submission form. Please remove any funding-related text from the manuscript.

**•Response: We have removed all the funding-related text from the manuscript.**

5. We note that Figure 1 in your submission contain map/satellite images which may be copyrighted. All PLOS content is published under the Creative Commons Attribution License (CC BY 4.0), which means that the manuscript, images, and Supporting Information files will be freely available online, and any third party is permitted to access, download, copy, distribute, and use these materials in any way, even commercially, with proper attribution. For these reasons, we cannot publish previously copyrighted maps or satellite images created using proprietary data, such as Google software (Google Maps, Street View, and Earth). For more information, see our copyright guidelines: http://journals.plos.org/plosone/s/licenses-and-copyright.

We require you to either (a) present written permission from the copyright holder to publish these figures specifically under the CC BY 4.0 license, or (b) remove the figures from your submission:

a. You may seek permission from the original copyright holder of Figure 1 to publish the content specifically under the CC BY 4.0 license.

**•Response: All figures are either produced by the authors or we have the express written permission of the author (Fig S1) to include and reproduce them in the article (permission uploaded into the system).**

**We have added in each Figure the source of the information (all freely available) and the software used to generate them.**

6. Please include captions for your Supporting Information files at the end of your manuscript, and update any in-text citations to match accordingly. Please see our Supporting Information guidelines for more information: http://journals.plos.org/plosone/s/supporting-information.

**•Response: Done. We have removed all figures from the manuscript and left only the captions in the text.**

**Additionally, we have included the following SI not present in the previous version:**

**S1 Table occurrences Trithemis kirbyi 2022 TOTAL.xlsx**

**S2 Table Trithemis kirbyi occurrences for modelling.xlsx**

**S3 Table Data Area-Thermal anomaly.xlsx**

**S1 Code R for Trithemis kirbyi analyses 2022.R**

7. We note that Supporting Information Figures 2 and 6 in your submission contain map/satellite images which may be copyrighted. All PLOS content is published under the Creative Commons Attribution License (CC BY 4.0), which means that the manuscript, images, and Supporting Information files will be freely available online, and any third party is permitted to access, download, copy, distribute, and use these materials in any way, even commercially, with proper attribution. For these reasons, we cannot publish previously copyrighted maps or satellite images created using proprietary data, such as Google software (Google Maps, Street View, and Earth). For more information, see our copyright guidelines: http://journals.plos.org/plosone/s/licenses-and-copyright.

We require you to either (a) present written permission from the copyright holder to publish these figures specifically under the CC BY 4.0 license, or (b) remove the figures from your submission:

a. You may seek permission from the original copyright holder of Supporting Information Figures 2 and 6 to publish the content specifically under the CC BY 4.0 license.

content specifically under the CC BY 4.0 license.

**•Response: As done for point 5 above, we confirm that all figures are either produced by the authors or we have the express written permission of the author (Fig S1) to include and reproduce them in the article (permission uploaded into the system).**

**We have added in each Figure the source of the information (all freely available) and the software used to generate them.**

**© 2023. This work is licensed under a CC BY 4.0 license.**

5. Review Comments to the Author

Please use the space provided to explain your answers to the questions above. You may also include additional comments for the author, including concerns about dual publication, research ethics, or publication ethics. (Please upload your review as an attachment if it exceeds 20,000 characters)

Reviewer #1: This is an impressive paper, very professional, outstanding and interesting for a broad audience, and I have no hesitation to recommend publication in its current version and congratulate the authors.

JS CARRION

**•Response: We would like to thank reviewer JS Carrión for his very positive comments about the article. We are glad that he found it a good contribution to PLOS ONE and to science.**

Reviewer #2: In the manuscript “Climate change as a driver of insect invasions: Dispersal patterns of a dragonfly species colonizing a new region”, Gil-Tapetado and collaborators used citizen-science data to model the potential distribution in Europe (as well as in the native range) of a recently arrived (neonative) dragonfly. They also assessed the potential role of different bioclimatic variables on its survival and spread in Europe and tested for a relation between range expansion and heat waves in the Iberian Peninsula in the period 2010-2022.

They found that this dragonfly will very probably spread along the European Mediterranean coast, as well as inland in Portugal, Spain, France, Italy. Interestingly, they found a relation between the annual rate of increase in the area occupied and the annual temperature anomaly (supposedly the summer heat waves, but see below).

I think that the manuscript is interesting and relevant. Furthermore, it deals with the effects of heat waves on animal spread, a subject that has been difficult to study on terrestrial ecosystems.

I have some major questions, mostly on some methodological issues:

1): Line 196 – specific analysis for the summer temperatures

Here the authors try to assess the role of the summer temperature peaks on dispersal speed and spread. However, they use the mean annual temperature change (annual thermal anomaly). This annual thermal anomaly necessarily includes the effects of mild winters, I guess. Is there in effect a very high correlation between summer temperature anomalies and annual temperature anomalies?

I mean – the authors themselves identified the minimum temperature of the coldest month as the most important variable in their distribution models. The dragonfly dispersal stage is the flying adult, and presumably it will fly in the summer, but I think there should be a discussion on the relative effects of adult flight/ nymph overwinter survival on the pattern of spread.

**•Response: The Minimum Temperature of the Coldest Month is highly correlated (0.96) with the Annual Mean Temperature. The anomaly occurs in all seasons of the year, but it is stronger in the winter (https://climate.nasa.gov/climate_resources/300/video-climate-spiral-1880-2022/), allowing the specimens that have migrated to northern and previously colder areas to establish more easily; in fact, the Minimum Temperature of the Coldest Month is becoming less of a limiting factor for the establishment and spread dispersion of *Trithemis kirbyi* to northern areas.**

2) Line 202 –the Mann-Whitney-Wilcoxon U test (a rank sum test) was used to compare increase in area (quadratic) with temperature anomaly (linear). They could have linearized the area. On line 252 these results are referred to as correlations.

**•Response: Thank you for the suggestion. We included now the results with the linearized area which indeed demonstrate the strong correlation between the variables.**

3) Line 203 – the explanation of how the authors calculated the maximum observed dispersal distance is unclear. The highest latitude point in consecutive years can occur at widely different longitudes, meaning that a short increase in latitude may refer to a very large or very small distance. So “distance between highest latitude points in consecutive year” is not correct; is it the length of the projection over the same longitude of the northernmost latitudes in consecutive years?

**•Response: We have modified the sentence to clarify that the “distance” is in the south-north direction only.**

4) Line 315 – “there is no niche shift” – where is this shown? There is a world of models analysing and measuring the niche shifts of invasive species in their new ranges. I don’t think this paper is analysing this issue.

**•Response: We agree with the reviewer. In fact, we are preparing a follow-up article that explores further these questions about the dragonfly, its annual dispersal and the niche conservatism. We have qualified the sentence by adding an “a priori” because it is true that in this article we have not demonstrated it empirically. However, we believe that looking at the distribution of *T. kirbyi*, it is not present in climatically very different areas from the Mediterranean basin, such as southern South Africa or North Africa, which is indeed a Mediterranean climate.**

**We have modified the phrase and added more context to make it clearer.**

Some more questions, referring to the text:

Introduction – from line 70 onwards, I question the need to build the introduction as if this manuscript would focus on competitive displacement of native species by T. kirbyi. It is out of scope with the goal of the manuscript. I would include the current trends in climate change at the beginning of the introduction, namely mentioning heat waves and current aridification / increase of minimum temperatures / reduction of the harsh conditions in winter

Line 97 – This is of the paragraphs I would move to the beginning of the introduction (it could be the second)

Line 103 - If the point is to present the objectives in this sequence, then rising temperatures and warming peaks must be addressed sooner in the introduction. And a bit more detailed (see the 2 previous comments)

**•Response: Regarding these three comments, we believe that the Introduction now maintains now a coherence that would be compromised by changing the order of the paragraphs. Changing their order would make it more difficult to follow our argument, and how the research question first arose.**

**We kindly ask the Editor to consider our request to keep the order of the paragraphs in the Introduction as it is now.**

Line 116- what is the study area? The entire distribution of the species whether in its native or neonative range, right? It is not clear throughout the manuscript and should be mentioned in this paragraph

**•Response: The study area is the whole range of the species as it is clear from figures 1 and S1, although the dispersal analysis focus in the spread of the species into Europe as indicated in the objectives of the study.**

Line 146 – correctly, the authors refer that one of the variables of each pair of correlated variables was removed. But what was the criteria of choice?

**•Response: We have added as small explanation in the text “…leaving the one perceived to have higher relative ecological importance and predictor effectiveness.**

Line 172 – a bit confusing and not explained before – so the occurrences of T. kirbyi from the Odonata Database of Africa were not used before? I guess only those occurrences from Africa but retrieved from iNaturalist were used, then? Only on line 225 the authors finally refer that the points from Odonata Database of Africa were not used at all in the modelling procedure.

So, correctly, the authors thought about using this different set of data to validate their models. This was not explained (the use of two separate sets of data for different goals) where it should have been (line 116 - Ocurrence data).

**•Response: Corrected, we have added in “M & M, Occurrence data, the sentence:**

**“Models were evaluated with occurrences from Odonata Database of Africa, which were not used at all in the modelling procedure.”**

Line 183 – western Mediterranean – the limits of this region are?

**•Response: We have included in the text “(the Mediterranean west of the Italian Peninsula and Sicily)” to clarify.**

Line 238 – Figure 3 is superfluous as a main figure – could be shown as supplemental data

**•Response: We strongly feel that this figure is crucial to follow our reasoning that the relation is strong, and that the minimum temperature of the coldest month is important for the distribution of the species.**

Line 262 – “that were previously unsuitable for the species” – a bit too overinterpreted. In spite of its velocity of expansion, the species could have juts been unable to fly directly to there.

**•Response: We do not think this is an overinterpretation. *Trithemis kirbyi* is able to spread in a radius of 400 km/year and it was already in northern Africa during the early 1990’. It is only in recent past years that it successfully colonized Europe (until Belgium now!), and we think that the rising of temperatures is the reason, not that it has not the ability to fly everywhere.**

Line 274 – Figure 3? It does not show anything about dryness

**•Response: Corrected**

Line 343 – “see Figure 2”? This Figure does not show increases in scarcity in the Sahara Desert

**•Response: Corrected**

Minor issues

Line 60 – “friends” (not necessary)

**•Response: Corrected**

Line 88 – of which only four (delete “,”)

**•Response: Done**

Line 124 – “than to less”

**•Response: Corrected**

Line 159 – considered them in

**•Response: Done**

Line 244 - ?as the crow flies?

**•Response: It’s an English expression that *used to say that a distance is measured in a straight line between two points or places.*** [**https://dictionary.cambridge.org/dictionary/english/as-the-crow-flies**](https://dictionary.cambridge.org/dictionary/english/as-the-crow-flies)

[**https://en.wikipedia.org/wiki/As_the_crow_flies**](https://en.wikipedia.org/wiki/As_the_crow_flies)

**We clarified this in the ms**

Line 259 – figure 5?

**•Response: Yes, it was a typo. Thank you.**
